# Longitudinal associations between problematic social media use and depressive symptoms in adolescent girls

**DOI:** 10.1016/j.pmedr.2019.100925

**Published:** 2019-06-21

**Authors:** Lennart Raudsepp, Kristjan Kais

**Affiliations:** a Institute of Sport Sciences and Physiotherapy, Faculty of Medicine, University of Tartu, 4 Ujula street, EE 51018 Tartu, Estonia; b Institute of Sport Sciences and Physiotherapy, University of Tartu, Tartu, Estonia

**Keywords:** Problematic social media use, Depressive symptoms, Adolescent girls

## Abstract

The primary aim of the current study was to examine longitudinal relation between problematic social media use (PSMU) and depressive symptoms in adolescent girls. Adolescent girls (n = 397) were assessed in three time points across two years. PSMU and depressive symptoms were subjectively assessed at three waves spaced 2 years apart. Latent growth models were used to test whether changes in PSMU were related to changes in depressive symptoms, and whether baseline PSMU predicted changes in depressive symptoms and vice versa. Results revealed baseline PSMU was positively associated with baseline depressive symptoms (β = 0.29, *p* < 0.01), and changes in PSMU were related to changes in depressive symptoms (β = 0.22, *p* < 0.05). In addition, baseline depressive symptoms were predictive of changes in PSMU (β = 0.23, *p* < 0.05), but baseline PSMU did not predict changes in depressive symptoms. These findings provide evidence of positive associations between increasing PSMU and depressive symptoms and suggest that interventions should target reduction of PSMU to prevent adolescents' mental health problems.

## 1 Introduction

Adolescence is a developmental period of heightened vulnerability for the onset of internalizing psychopathology, particularly major depression and depressive symptoms (McLaughlin and King, 2015). It is estimated that up to 20% of children and adolescents experience depressive symptoms and during the few years from early to mid-adolescence, the level of depressive symptoms sharply increases (Avenevoli et al., 2015). Additionally, adolescent depressive symptoms and depression commonly persist, recur, and continue into adulthood and portend a wide range of negative consequences across the life-course, including poor psychosocial functioning (Ellis et al., 2017).

Depressive symptoms are more common in adolescent females and the presence of depressive symptoms and disorders intensively increases in girls between the ages of 13 and 15 (Gomez-Baya et al., 2017; McLaughlin and King, 2015). It is therefore of great interest to understand why the level of girls' depressive symptoms is increasing during adolescence, to identify possible risk and protective factors, and to develop effective prevention approaches (Yoon et al., 2019). It has been demonstrated in recent research that increasing social media use is one important factor affecting adolescents' mental health, and that social media use may have more adverse psychological impact on girls than on boys (McRae et al., 2017). For example, Neira and Barber (2014) found that female adolescents who had a social media profile reported significantly higher levels of depressed mood, and lower self-esteem, than female youth without a social media profile. When used at moderate level, social media use is more likely to bring benefits because it offers opportunities to develop new friendships and to maintain existing ones, but only when there is an overlapping of the offline and online social worlds (Nowland et al., 2018).

Besides functional social media use, there is an increasing number of adolescents experiencing negative consequences due to problematic social media use (PSMU, Shensa et al., 2017). PSMU has been characterized by an excessive concern about social media, being driven by a motivation to use social media, and devoting so much time and effort to social media use that it limits other social activities, studies, interpersonal relationships, mental health and well-being (Andreassen, 2015). PSMU has characterized as a maladaptive pattern of social media use (Shensa et al., 2017) and there is a growing scientific evidence base to suggest PSMU may lead to symptoms traditionally associated with substance-related addictions (Andreassen, 2015). For example, Salmela-Aro et al. (2017) found reciprocal longitudinal association between excessive internet use and school burnout in 12–18-year olds Finnish adolescents. Additionally, cross-cultural research including 10,930 adolescents from six European countries showed that excessive use of social media was related to internalizing problems, decreased academic performance and higher risk of overweight/obesity (Tsitsika et al., 2014, Tsitsika et al., 2016). The prevalence of PSMU varies among adolescent populations ranging from 4,5% in Hungary (Bányai et al., 2017) to 25,5% in China (Li et al., 2018), although only the study by Bányai et al. (2017) used a nationally representative sample.

Empirical studies examining the prospective association between PSMU and depressive symptoms in adolescents are relatively limited and with mixed results (McRae et al., 2017). For example, two studies reported that adolescents' depressive symptoms and PSMU did not exhibit significant relations over a period of 6 (van den Eijnden et al., 2008) and 12 months (Brailovskaia and Margraf, 2017). In contrast, two recent studies found a bidirectional association between PSMU and depressive symptoms in male adolescents only (Houghton et al., 2018) and in adolescents who were persistently depressed at baseline (Li et al., 2018). Furthermore, no studies investigate dynamic associations of changing depressive symptoms and PSMU over time using latent growth modeling. Latent growth curve models enable an estimation of the within-person fluctuations of the trajectory of change over time and an assessment of how the changes in depressive symptoms and PSMU may be interrelated (Bollen and Curran, 2006). Clarifying these associations in adolescent females remains particularly important, considering that depressive symptoms (McLaughlin and King, 2015) and PSMU (Andreassen et al., 2017) are known to undergo dramatic changes during this period as compared to other stages of development. Given the few researches have examined the changes and bidirectional associations of PSMU and depressive symptoms among adolescent girls, the aim of this study was to examine the longitudinal relation between PSMU and depressive symptoms.

## 2 Methods

### 2.1 Participants and study design

An initial sample of 397 female secondary school students (Grades 6 and 7) with a mean age of 12,6 ± 0.7 years, from six schools in Tartu city, Estonia, completed measures of depressive symptoms and PSMU over a two-year period: September 2016 (T1), September 2017 (T2) and September 2018 (T3). All 14 middle public schools of city were invited to participate and the principals of six schools agreed to participate. Seven schools refused to participate due to different reasons, mainly due to overload of students with different projects (e.g. research projects) and 1 school was Russian. All 6 participating schools located in the city suburbs where the family's socioeconomic characteristics (e.g., mean family's income per year = 30,000–34,999 euro) was similar as indicated by the data from Estonian Agency of Statistics. Researcher-school agreement regarding voluntary participation for conducting the measurements at a regular interval was arranged. To reduce selection bias, girls were randomly selected from a class roster, and asked to participate in this study. Only those for whom written parental consent was obtained in both the six and seventh grades were included in this study. About half of participants were in sixth grade (46,6%) and about half were in seventh grade (53,4%) at T1.

Investigators from this study visited the selected schools and gave an overview of the study to girls, and sent information letters to their parents. Participants completed the paper and pencil instruments at one meeting in a quiet classroom conditions in the presence of the member of a research team. It took approximately 20 min to complete all the questionnaires. Similar data collections procedures were followed at T1, T2 and T3 waves. At baseline, there were a total of 397 girls who completed the questionnaires. There were 365 adolescents (91,9%) in interim measurement and 343 adolescents (86,3%) in follow-up measurement who provided complete data. All procedures were also approved by the Medical Ethics Committee of Tartu University.

### 2.2 Measures

#### 2.2.1 Problematic social media use

Bergen Social Media Addiction Scale (BSMAS, Andreassen et al., 2017) was used to assess participants' PSMU. BSMAS is a modified version of the previously validated Bergen Facebook Addiction Scale (Andreassen et al., 2012). The adapted scale comprised a wording change that replaced “Facebook” with “Social Media” in each item, with social media defined in the scale instructions as “Facebook, Twitter, Instagram etc.”. BSMAS has been composed of 6-items reflecting the six core addiction elements (Griffiths, 2013) i.e., salience (“Spent a lot of time thinking about social media or planned use of social media”), mood modification (“Used social media in order to forget about personal problems”), tolerance (“Felt an urge to use social media more and more”), withdrawal (“Become restless or troubled if you have been prohibited from using social media”), conflict (“Used social media so much that it has had a negative impact on your studies”), and relapse (“Tried to cut down on the use of social media without success”). Each item is answered on a five-point Likert scale, with score ranging from 1 (very rarely) to 5 (very often). The BSMAS score was obtained by summarizing the responses of all six items. The scale was translated to Estonian and then back-translated by independent translators. The back-translation was then compared with the original scale and adjustments were made as necessary. The BSMAS is a psychometrically valid scale (Bányai et al., 2017). The Cronbach's alpha of the translated BSMAS was 0.78 in the present sample at T1. We next estimated a uni-dimensional confirmatory factor analysis (CFA) model for BSMAS. Results of these analyses indicated that the model did fit well (RMSEA = 0.06, CFI = 0.95, TLI = 0.93) using T1 data.

#### 2.2.2 Depressive symptoms

Depressive symptoms were assessed using the Centre for Epidemiological Depression Scale (CES-D, Radloff, 1977). This instrument demonstrated good reliability and factorial validity for the measurement of depressive symptoms (Radloff, 1977). The CES-D is a 20-item measure assessing symptoms of depression with items phrased as self-statements (e.g. “I felt hopeful about the future”). Ratings were based on a 4-point Likert scale ranging from 0 (rarely or none of the time) to 3 (most or all of the time), yielding a total score ranging from 0 to 60, in which a higher score indicates more severe depressive symptoms. Respondents rated how frequently each item applied to them over the course of the past week. The scale was translated to Estonian and then back-translated by two independent translators. The back-translation was then compared with the original CES-D and adjustments were made as necessary. The Cronbach's alpha of the translated CES-D was 0.81 in the present sample at T1. Finally, we estimated a uni-dimensional confirmatory factor analysis (CFA) model for CES-D. Results of these analyses indicated that the model did fit well (RMSEA = 0.05, CFI = 0.96, TLI = 0.95) using T1 data.

#### 2.2.3 Control variables

Participants' age was used as a control variable in LGM models.

### 2.3 Statistical analysis

Descriptive statistics (means, standard deviation) of the study variables were calculated. Pearson product-moment correlation coefficients were calculated to assess the relationships between variables. We estimated the effect sizes over the 2-year period for PSMU and depressive symptoms (0,2, 0,5 and 0,8 for small, moderate, and large effects, respectively, Cohen, 1992). Latent growth modeling (LGM; Bollen and Curran, 2006) was used to determine longitudinal relation between PSMU and depressive symptoms. In LGM models, intercepts represent the baseline level for each variable while slopes represent the change over time. First, we estimated the trajectories of change in PSMU and depressive symptoms using separate unconditional models. Linear growth was reflected by fixing the paths from the latent linear factor to PSMU variables as 0, 1, 2. The mean intercept and linear factor were assessed to determine the average initial PSMU and the average rate of change in PSMU over time. The variance of the intercept and linear factor were examined to determine individual differences in initial PSMU and the rate of change in PSMU over time. The linear growth model for depressive symptoms was the same as that described above for PSMU. Next, individual growth models of PSMU and depressive symptoms were combined to examine how the slopes of PSMU and depressive symptoms interrelated. The intercept of one growth model was also expected to predict slope of the second growth model, therefore bidirectional predictions were added from the intercept to the slope for each of the models. Full information maximum likelihood estimation with robust standard errors was used to account for missing data and the non-normality of variables. Depressive symptoms had an approximate normal distribution whereas PSMU had a positive skew. Model fit was assessed using the chi-square, comparative fit index (CFI), Tucker-Lewis index (TLI) and root mean square error of approximation (RMSEA). An acceptable fit of the data is indicated by values ≥0.90 for the CFI and TLI, and value ≤0.08 for the RMSEA (Hu and Bentler, 1999). All preliminary analyses were conducted in SPSS version 23.0 and LGM was conducted in Amos 23.0.

## 3 Results

95% of the participants had cell phone and all participants had access to computer and internet at home. The percentage of participants with clinically relevant levels of depressive symptoms, i.e. with CES-D scores ≥20 (Vilagut et al., 2017), increased from 3.7% to 5.6% between T1 and T3. The proportion of girls who were classified as at-risk of PSMU (≥19, Bányai et al., 2017) increased from 7.7% at T1 to 9.3% at T3.

### 3.1 Correlation analysis

A correlation matrix including means and standard deviations is presented in Table 1. Analyses revealed the positive and statistically significant bivariate relationships between PSMU and depressive symptoms at T1, T2 and T3 (*r* = 0.17–0.43, *p* < 0.05–0.01).

**Table 1:** Descriptive statistics and correlations among variables.

Measure	M	SD	1	2	3	4	5	6	7
1 PSMU T1	11.5	1.6	–	0.43⁎⁎	0.37⁎⁎	0.23⁎⁎	0.18⁎	0.15⁎	0.06
2. PSMU T2	11.9	1.5		–	0.34⁎⁎	0.25⁎⁎	0.27⁎⁎	0.20⁎⁎	0.04
3. PSMU T3	12.6	1.5			–	0.22⁎⁎	0.17⁎	0.30⁎⁎	0.08
4. DS T1	15.9	1.9				–	0.38⁎⁎	0.33⁎⁎	−0.09
5. DS T2	16.4	1.8					–	0.37⁎⁎	−0.06
6. DS T3	16.9	1.8						–	−0.05

PSMU, problematic social media use; DS, depressive symptoms.

⁎ *p* < 0.05.

⁎⁎ *p* < 0.01.

### 3.2 Estimating growth in PSMU

There was a linear increase in PSMU over time. The effect size for 2-year change (*d* = 0.15) was small in magnitude. The unconditional model provided a good close fit to the data (χ^2^ (1) = 78.46; RMSEA = 0.054), (*CI*_*95*_ = [0.039–0.068]; TLI = 0.961; CFI = 0.967), and there was a significant, linear increase in PSMU over time (mean slope = 0.21, SE = 0.08, *p* < 0.05). The linear change for PSMU was not related to the initial level of PSMU (*r* = 0.10, *p* > 0.05). This indicates that participants with greater initial PSMU had no greater increase in PSMU than participants with lower baseline PSMU. Significant variations (*p* < 0.05) were found for the initial level and linear change, which suggest that the initial level and change in PSMU differed significantly from one participant to another.

### 3.3 Estimating growth in depressive symptoms

A linear increase in depressive symptoms was observed over time. The effect size for 2-year change (*d* = 0.10) was small in magnitude. The unconditional model provided a good close fit to the data (χ^2^ (1) = 71.62; RMSEA = 0.059), (*CI*_*95*_ = [0.043–0.075]; TLI = 0.956; CFI = 0.960), and there was a significant linear increase in depressive symptoms over time (mean slope = 0.13, SE = 0.06, *p* < 0.05). The linear change for depressive symptoms was positively related to the initial level of depressive symptoms (*r* = 0.25, *p* < 0.05). This indicates that participants with greater initial depressive symptoms had greater increase in depressive symptoms than participants with less initial depressive symptoms. Significant variations (*p* < 0.05) were found for the initial level and linear change, which suggest that the initial level and change in depressive symptoms differed significantly from one participant to another.

### 3.4 Parallel process model

To examine the relation between the trajectories of PSMU and depressive symptoms, a parallel LGM was tested first since we expected growth in each process to be correlated. This model fit the data well (χ^2^ (36) = 78.11; RMSEA = 0.043, (*CI*_*95*_ = [0.035–0.051]); TLI = 0.971; CFI = 0.983). There was a significant association between initial PSMU and initial depressive symptoms (*r* = 0.29, *p* < 0.01), indicating that participants who started with a higher PSMU had significantly higher depressive symptoms. The covariance between the PSMU change and depressive symptoms change terms was also statistically significant (*r* = 0.22, *p* < 0.05), indicating that participants with greater increases in one construct tended to have greater increases in other construct over time. In terms of bidirectional associations, the path from initial depressive symptoms to the change in PSMU was significant (*β* = 0.23, *p* < 0.05), indicating that participants who started with higher depressive symptoms tended to have steeper increases in PSMU. Additionally, the intercept of PSMU did not predict the slope of depressive symptoms (*β* = 0.06, *p* > 0.05). This indicates that participants with greater initial PSMU had no greater increase in depressive symptoms across time than participants with lower baseline PSMU.

**Fig. 1:**
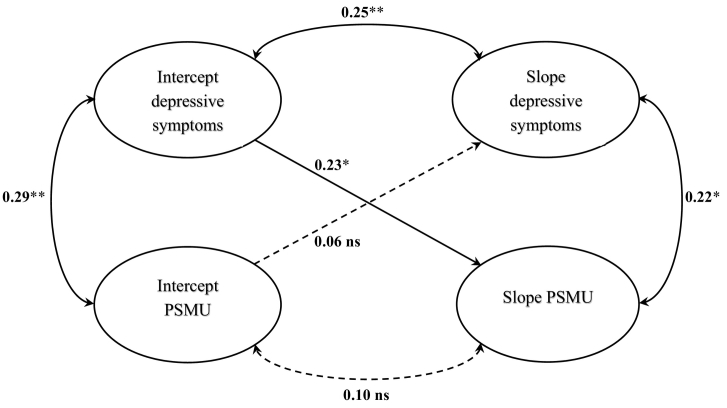
Parallel process latent growth model for depressive symptoms and problematic social media use. Standardized coefficients are presented. PSMU, problematic social media use. *p < 0.05; **p < 0.01.

When adding age as covariate, model fit remained good and results were consistent with the unadjusted model (χ^2^ (43) = 87.38; RMSEA = 0.046, (*CI*_*95*_ = [0.038–0.054]); TLI = 0.965; CFI = 0.973). Participants' age was significantly related to intercept of PSMU (*β* = 0.16, *p* < 0.05). Results from parallel process LGM are presented in Fig. 1.

## 4 Discussion

The present study makes distinctive contributions to the literature by simultaneously examining the parallel longitudinal associations between PSMU and depressive symptoms in adolescent females over a 2-year period. Results indicated that both PSMU and depressive symptoms increased over two years and changes on PSMU were associated with changes in depressive symptoms. Additionally, higher baseline depressive symptoms predicted steeper increases in PSMU, but initial PSMU did not predict change in depressive symptoms over time.

The results of LGM showed that an increase in adolescent girls' PSMU was related with an increase in depressive symptoms over two years. To our knowledge, our study is the first to examine parallel changes in adolescent girls PSMU and depressive symptoms using LGM. A similar modeling approach was used by Ciarrochi et al. (2015) who found that slopes of compulsive internet use and poor mental health (e.g., depressive symptoms, negative affect) were positively associated (*r* = 0.62), indicating that increases in adolescents' compulsive internet use were associated with increase in poor mental health. A major advantage of LGM is that it considers both between-person and within-person variability when analyzing the change of construct of interest (Bollen and Curran, 2006). As in any longitudinal study, however, the period of the time lags between each wave warrants discussion, because the effects of PSMU on mental health would be expected to take time to emerge across adolescence. Therefore, longitudinal studies with longer follow-up than used in the present study, are needed.

A growing body of recent research suggests that there is a bidirectional relationship between adolescents' online social networking addiction and depression (Li et al., 2018), problematic internet use and depressive symptoms (Gamez-Guadix, 2014) and screen use and depressive symptoms among those adolescents who experienced increases in depressive symptoms over time (Houghton et al., 2018). No evidence for a bidirectional association between PSMU and depressive symptoms was identified in the current study. Additionally, our results indicated that higher baseline depressive symptoms predicted steeper increases in PSMU over time. This finding agreed with that reported in the longitudinal study of Gamez-Guadix (2014), who found that depressive symptoms predict an increase in problematic internet use after 1 year. Additionally, recent research by Oberst et al. (2017) found that only for females, depressive symptoms significantly predicted negative consequences of social networking site use. It might be possible that, for girls, higher levels of depressive symptoms may result in higher social media usage, implicating cyclical relationships in that depressive symptoms may exacerbate negative consequences due to social media use, which may then negatively impact upon perceived depression symptoms (Kuss and Griffiths, 2017).

Contrary to the present findings, three recent longitudinal studies found that compulsive internet use (Ciarrochi et al., 2015), online social networking addiction (Li et al., 2018) and use of mobile phones (Bickham et al., 2015) was a consistent predictor of adolescents' poor mental health variables (e.g., depressive symptoms, depression and negative affect). For example, Li et al. (2018) found that adolescents who were persistent or emerging online social networking addiction have an increased risk of developing depression compared with those who were not addictive to social networking use at baseline. Differences between studies in sample characteristics, measurement methods, length of follow-up and whether PSMU or typical social media use was assessed, may partly explain the inconsistent findings. Further, relatively modest unidirectional effects of increased depressive symptoms on PSMU may accumulate over time and stronger associations might be seen when longer duration follow-up will be used.

The results about the underlying mechanisms explaining the longitudinal relationship between depressive symptoms and PSMU are scarce (Ciarrochi et al., 2015). It is possible that girls with higher depressive symptoms may prefer social media communication to face-to-face communication upon perceiving online communication to be more secure and less threatening, as well as a means for regulating their negative moods (Kraut et al., 1998). It is also likely that adolescent girls with higher levels of depressive symptoms may activate less effective coping strategies (e.g., seeking social support), which in turn could increase the likelihood of PSMU (Gamez-Guadix, 2014). Another plausible scenario is that the longitudinal association between PSMU and depressive symptoms is still bidirectional, although our findings did not support this. Future studies involving representative adolescent samples of both females and males, adopting different time lags between measurement waves, and involving latent-class trajectory modeling approach, are needed. Additionally, qualitative methods have an important part to play in understanding the phenomenon of mental health impact of PSMU in adolescent population. Prevention efforts targeted at identifying depressed youths should also consider screening for amount and frequency of social media use. Finally, it is also of fundamental importance to analyze what kinds of activities are being engaged in social media, rather than the medium through which these activities are engaged in (Kuss and Griffiths, 2017).

### 4.1 Strengths

The current paper provides a relevant addition to the literature in three ways. First, data across three time points were collected and analyzed using a novel statistical technique to the analysis of changes and longitudinal associations between PSMU and depressive symptoms. Second, we showed that the dynamics of upward spirals in depressive symptoms occurred together with upward spirals in PSMU. Third, this is the first longitudinal study on PSMU and depressive symptoms conducted with Estonian adolescents.

### 4.2 Limitations

There is number of limitations that must be considered when interpreting our findings. First, adolescent females were followed for 2 years only, thus potential long-term effects between PSMU and depressive symptoms were not explored. A greater number of data points, longer time intervals, and timing of assessment of social media use should be considered to model developmental trajectories and parallel associations of change. Second, the relatively small sample size reduces the degree to which we can be confident that the results generalize to a broader population of adolescent girls. Furthermore, PSMU and depressive symptoms were assessed using self-report. Also, we relied exclusively on total score derived from BSMAS but did not focused on several important aspects of social media use such as: a) what kinds of social media activities and how frequently were engaged, and b) valence of these social media experiences. For example, recently Primack et al. (2018) showed that only negative but not positive experiences on social media were strongly associated with higher depressive symptoms in university students. Finally, there might be other unmeasured variables related to development, environment, or genetics that explain changes in both PSMU and depressive symptoms (Ciarrochi et al., 2015). For example, the development of adolescents' depressive symptoms is influenced by parental factors, such as parental depression and parent/child relationship (Bickham et al., 2015), and personality traits and facets, such as neuroticism and low conscientiousness and sociability (Goldstein et al., 2018).

## 5 Conclusions

This study's findings indicate that PSMU and depressive symptoms were associated across two years – increase in the trajectory of PSMU was associated with an increase in the trajectory of depressive symptoms. Higher baseline depressive symptoms predicted steeper increase in PSMU over time. Further longitudinal research is needed to examine whether the associations between adolescents' PSMU and mental health varied based on initial status and developmental patterns of both social media use and depressive symptoms using more refined measures for social media use.

## Declaration of Competing Interest

None.
